# DEAD-box helicase 56 functions as an oncogene promote cell proliferation and invasion in gastric cancer via the FOXO1/p21 Cip1/c-Myc signaling pathway

**DOI:** 10.1080/21655979.2022.2084235

**Published:** 2022-06-19

**Authors:** Jiancheng Wang, Ye Wang, Junfu Wang, Siwen Zhang, Zhu Yu, Kaitian Zheng, Zhao Fu, Congjun Wang, Weijia Huang, Junqiang Chen

**Affiliations:** a Department of Gastrointestinal Gland Surgery, the First Affiliated Hospital of Guangxi Medical University, Guangxi Medical University, Nanning, Guangxi, China; b Guangxi Key Laboratory of Enhanced Recovery after Surgery for Gastrointestinal Cancer, The First Affiliated Hospital of Guangxi Medical University, Nanning, Guangxi, China; c Department of General Surgery, The First Affiliated Hospital of Nanchang University, Nanchang, China

**Keywords:** DDX56, gastric cancer, FOXO1/p21 Cip1/c-Myc signaling pathway, proliferation, apoptosis

## Abstract

DEAD-box helicase (DDX) family exerts a critical effect on cancer initiation and progression through alternative splicing, transcription and ribosome biogenesis. Increasing evidence has demonstrated that DEAD-box helicase 56 (DDX56) is over-expressed in several cancers, which plays an oncogenic role. Till the present, the impact of DDX56 on gastric cancer (GC) remains unclear. We conducted high-throughput sequencing (RNA-seq) to demonstrate aberrant DDX56 levels within 10 GC and matched non-carcinoma tissue samples. DDX56 levels were detected through qRT‐PCR, western blotting (WB) and immunochemical staining in GC patients. We conducted gain- and loss-of-function studies to examine DDX56’s biological role in GC development. In vitro, we carried out 5‑Ethynyl‑2‑deoxyuridine (EdU), scratch, Transwell, and flow cytometry (FCM) assays for detecting GC cell growth, invasion, migration and apoptosis. Additionally, gene set enrichment analysis (GSEA), WB assay, and Encyclopedia of RNA Interactomes (ENCORI) were carried out for analyzing DDX56-regulated downstream genes and signaling pathways. In vivo, tumor xenograft experiment was performed for investigating how DDX56 affected GC development within BALB/c nude mice. Functionally, DDX56 knockdown arrested cell cycle at G1 phase, invasion and migration of AGS and MKN28 cells, and enhanced their apoptosis. Ectopic DDX56 expression enhanced the cell growth, migration and invasion, and inhibited apoptosis. Knockdown of DDX56 suppressed GC growth in the tumor models of BALB/c nude mice. Mechanistically, DDX56 post-transcriptionally suppressed FOXO1/p21 Cip1 protein expression, which could activate its downstream cyclin E1/CDK2/c-Myc signaling pathways. This sheds lights on the GC pathogenic mechanism and offers a potential anti-cancer therapeutic target.

## Graphical abstract

**Figure:**
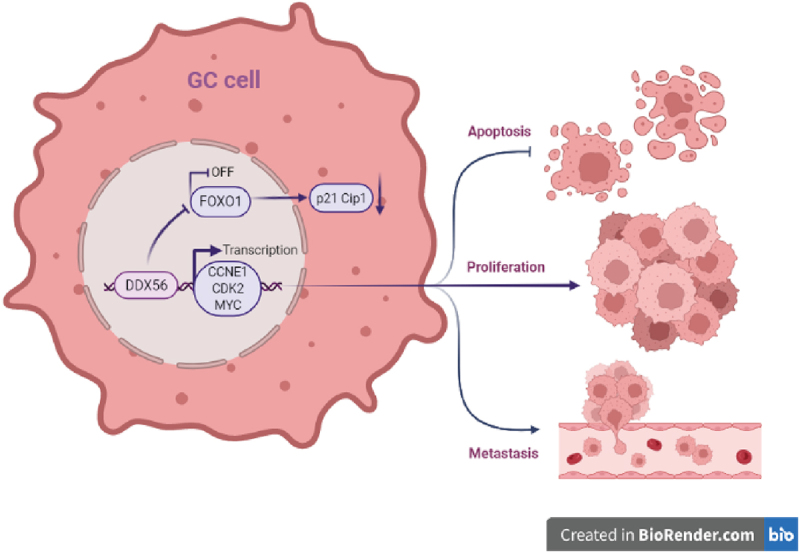

## Highlights

- High expression of DDX56 is associated with poor prognosis of GC patients.
- Ectopic DDX56 expression enhances the GC cell growth, migration and invasion, and inhibited apoptosis.
- DDX56 promotes the progression of GC through the FOXO1/p21 Cip1/c-Myc signaling pathway.

## Introduction

More than one million people across the world are diagnosed with gastric cancer (GC) every year [1]. GC morbidity and mortality rates show a declining trend worldwide in the last decade, accounting for the 3^rd^ leading cause of cancer-associated mortality and ranking the 5^th^ place in term of its morbidity worldwide [2]. More than 40% of new cases and deaths in China are attributed to GC annually [3]. Apart from that, a lot of factors are considered to be related to the dismal prognosis. The essential factors are inadequate early prediction, high degree of malignancy, and a lack of individualized therapeutic intervention [4]. As a result, it is urgent to further explore the mechanism of GC occurrence and development at molecular level, so as to formulate the effective treatments.

RNA helicases play essential roles in transitionally, transcriptionally and post-translationally regulating cell cycle regulator levels [5]. DEAD (Asp-Glu-Ala-Asp)-box RNA helicases represent the greatest largest group with 57 RNA helicases in human [6]. The deregulation of DEAD-box is related to tumor occurrence and development, such as cell growth, invasion and migration [7]. Existing evidence indicates that DDX5, DDX27, and DDX39 played a major role as oncogenes in gastric cancer and hepatocellular carcinoma (HCC). Recent in vitro studies have strongly suggested that DDX5 and DDX27 knockdown suppressed GC cell proliferation [8] and apoptosis [9]. In addition, DDX39 knockdown inhibited hepatocellular carcinoma (HCC) cell progression via the activation of Wnt/β-catenin pathway [10]. However, not all members of the DDX family have a positive role in promoting cancer. Some studies have reported that the expression of DDX20 was elevated in GC [11] and HCC [12] cells, which significantly inhibited cancer progression and improved patient prognosis. Recent studies targeting DDX56 in cancer is still in its infancy. Relevant studies have pointed out that DDX56 promoted cancer progression in colorectal cancer (CRC) [13], osteosarcoma cells [14] and squamous cell lung carcinoma [15]. Unfortunately, it remains largely unclear regarding DDX56’s biological functions in GC and its mechanisms.

The transcription factor (TF) family of forkhead box (FOX) has pleiotropic functions in physiology and physiopathology, such as cell cycle regulation, aging, tumor suppression, multiple apoptotic activities, autophagy and oxidative stress tolerance [16,17]. Most studies have demonstrated that the dysfunction of the FOXO1 signaling pathway is associated with numerous complex molecular cascades in cancer pathophysiology [18,19]. The cyclin-dependent kinase inhibitor p21 Cip1, which serves as a critical downstream effector of FOXO1, is one of the most studied CDK inhibitors and it regulates cell proliferation, cell motility, and apoptosis [19,20]. The p21 Cip1 can bind to CDK2, leading to the inactivation of CDK2/cyclin E1 at the G1-S phase DNA damage checkpoint, thereby arresting cells at the G1-S phase during DNA repair [21]. The recent study shows that FOXO1 induces irreversible G1 arrest and cellular senescence through the PR-B/FOXO1/p21 Cip1 axis in endometrial cancer [22]. Previously studies should be recognized that FOXO1 can promote the apoptosis signaling by the activation or repression of apoptosis-related genes (ARGs) [18,23]. Moreover, FOXO1 reduces the Bcl-2/BAX ratio induced by curcumin in pancreatic cancer [24].

In this study, we identified DDX56 by high-throughput sequencing in order to explore those differentially expressed genes between GC and adjacent normal gastric tissues. Besides, the biological functions of DDX56 within GC cell lines (AGS, MKN28) were analyzed, which indicated that DDX56 enhanced cell growth and aggressive phenotypes, while inhibiting in-vitro apoptosis of cells. Additionally, we also constructed the nude mouse model of xenograft to determine the effect of DDX56 on GC cell proliferation in vivo. Mechanistically, we proposed that the FOXO1/p21 Cip1/c-Myc signaling pathway was involved in the regulation of DDX56.

## Materials and methods

### Patients and tissue samples

In this study, we acquired GC samples and matched non-carcinoma samples in 70 GC cases divided into three cohorts at the First Affiliated Hospital of Guangxi Medical University (Guangxi, China). Cohort 1 included 10 patients who underwent surgery from January 2019 to December 2019, and their tumors were collected for high-throughput sequencing. Cohort 2 consisted of 30 patients undergoing surgery between June 2019 and December 2020. Their tumors were collected for histological studies. Cohort 3 involved 30 patients who received surgery from June 2013 to December 2013, and their tumors were adopted for immunohistochemical (IHC) analysis.

The collected tissue samples were stored in the RNA Keeper Tissue Stabilizer (R501-01; Vazyme, Nanjing, China), put into an icebox and transferred to the −80°C refrigerator until RNA isolation was performed. All patients were treated without radiotherapy or chemotherapy before surgery. The Ethics Review Committee of the First Affiliated Hospital of Guangxi Medical University approved the study protocol. Patients provided the informed consent for tissue specimen collection. This research followed all the guidelines outlined in the Declaration of Helsinki.

### Immunohistochemical analysis and scoring

Immunohistochemical specimens were fixed in 10% formaldehyde, which were then embedded in wax blocks. This study obtained anti-DDX56 antibody in Santa Cruz Biotechnology (5A7: sc-101,018, Santa Cruz, CA) and Ki-67 polyclonal antibody in Proteintech Group (No. 26,593-1-AP, ProteinTech, Wuhan, China). Following manufacturer’s instruction, the endogenous peroxidase blocking reagent (SP-9000, Zhongshan Golden Bridge, Beijing, China), goat serum (SP-9000, Zhongshan Golden Bridge), anti‐DDX56 antibody, Ki-67 polyclonal antibody, goat anti-mouse IgG (SP-9000, Zhongshan Golden Bridge), goat anti-rabbit IgG (SP-9000, Zhongshan Golden Bridge), HRP-labeled avidin working fluid (SP-9000, Zhongshan Golden Bridge) and Diaminobenzidine (DAB; ZLI-9018, Zhongshan Golden Bridge) were added in a regular sequence. Finally, the tissue sections were stained, and then a light microscope (Olympus Corporation, Tokyo, Japan) was employed for observation. Tumor histology was independently reviewed by an experienced pathologist. The DDX56 levels were rated as 0–3, indicating negative, low, moderate, and high intensities, respectively. The scores regarding the extent of staining were 0 (0%), 1 (1–25%), 2 (26–50%), 3 (51–75%), and 4 (76–100%). In addition, a score of >4 was suggested as high.

### Cell culture

This study obtained the human GC cells (AGS, HGC27, and MKN28), and normal human gastric epithelial Ges-1 cells from the Institute of Biochemistry and Cell Biology (Shanghai Institute of Biological Sciences, Chinese Academy of Sciences). AGS cells were cultured in Ham’s F12 medium (Procell Life Science & Technology Co., Ltd., Wuhan, China) with 10% fetal bovine serum (FBS; Gibco, Shanghai, China), streptomycin (50 ug/ml), and penicillin (50 U/ml) (Solarbio, Beijing, China). With 10% FBS (Gibco) and penicillin–streptomycin (Solarbio), HGC27, and MKN28 cells were cultured in RPMI-1640 supplemented (Gibco). Ges-1 cells were cultured in DMEM high glucose medium (Gibco), including 10% FBS (Gibco) and penicillin–streptomycin (Solarbio). In line with specific protocols, we maintained cells within the humid incubator under 37°C and 5% CO_2_ conditions.

### Construction of DDX56 cell lines with plasmid transfection and lentivirus-based plasmid infection

The negative control (NC) plasmid, silencing plasmid, empty control plasmid, and overexpression plasmid of DDX56 were purchased from GeneCopoeia (Guangzhou, China). Lipofectamine^TM^ 3000 Transfection Reagent (Invitrogen, USA) was adopted for transient transfection in AGS and MKN28 cells in line with specific protocols. In order to silence DDX56 expression, these two cell lines were transfected with DDX56-silencing plasmid (HSH105032-LVRU6MP-c; sh-DDX56) or NC plasmid (CSHCTR001-LVRU6MP; NC-DDX56). Additionally, cells were transiently transfected with DDX56-overexpressing plasmid (EX-T0449-Lv206; OE-DDX56) or empty control plasmid (pEZ-Lv206; Vector). To generate the AGS cell line with stable DDX56 knockdown, cells were transfected with shRNA-DDX56-LV (sh-DDX56-LV) or scramble-DDX56-LV (NC-DDX56-LV) lentiviral particles. The total RNA and cellular protein of DDX56 in transfected cells were extracted for subsequent experiments.

### RNA isolation and Quantitative real-time polymerase chain reaction (qRT-PCR)

In brief, we isolated total tissue and cellular RNAs using the NucleoZOL Kit (Macherey-Nagel, Düren, Germany). Subsequently, total RNA (1000 ng) was prepared into cDNA with the HiScript III RT SuperMix for qPCR (R323-01, Vazyme, Nanjing, China) through reverse transcription, followed by preservation under −80°C. Using ChamQ Universal SYBR qPCR Master Mix (Q711-02, Vazyme), this study then conducted quantitative RT-PCR with the 7500 Real-time PCR system (ThermoFisher, USA). The 2-ΔΔCt method was applied in calculating relative gene levels as fold-change (FC), followed by normalization based on GAPDH expression. In quantitative QRT-PCR, the primers used were shown below: DDX56, 5’-CCGCTTATGCTATTCCGATGC-3’ (F), 5’-GCTCCTTGGTAGGAACAAGAACA-3’ (R); GAPDH, 5’-GGAGCGAGATCCCTCCAAAAT-3’ (F), 5’-GGCTGTTGTCATACTTCTCATGG-3’ (R). The normalized value of mRNA level was set to 1 for cells transfected with control plasmid.

### Western blotting (WB) assay

The whole cell lysates were extracted from tissues, Ges-1, AGS, and MKN28 cells using RIPA lysis buffer containing PMSF and phosphatase inhibitor (P0100, Solarbio). Protein content was measured by BCA Protein Detection kit (P0012S, Beyotime Biotechnology, Shanghai, China) to equalize protein loading. After separation by electrophoresis, proteins were transferred onto polyvinylidene fluoride (PVDF) membranes (Merck Millipore, Darmstadt, Germany), which were later blocked using 5% skim milk for 30–60 min. Then, we further incubated PVDF membranes based on DDX56 (1:200; 5A7; sc-101,018, Santa Cruz, CA), Bcl-2 (1:1000, No. 26,593-1-AP, ProteinTech), BAX (1:5000, No. 60,267-1-Ig, ProteinTech), FOXO1 (1:1000, No. 18,592-1-AP, ProteinTech), p21 Cip1 (1:1000, No. 10,355-1-AP, ProteinTech), CDK2 (1:1000, No. 10,122-1-AP, ProteinTech), and GAPDH (1:2000, No. HRP-60004, ProteinTech). After washing with TBST, PVDF membranes were subsequently incubated using HRP-conjugated secondary antibody (ProteinTech) for a 1-h period under ambient temperature. The ECL chemiluminescence kit (Thermo Fisher Scientific, Waltham, MA, USA) was adopted for visualizing blots. Image J (1.8.0, NIH, USA) was utilized for performing image analysis. Protein expression was normalized to GAPDH and the mean value of the group was plotted in a bar chart.

### 5‑Ethynyl‑2‑deoxyuridine (EdU) assay

The cells were co-incubated with EdU working solution (C0071S, Beyotime, Shanghai, China) for a 2-h period under 5% CO_2_ and 37°C conditions. Subsequently, using Alexa Fluor 488 (Beyotime), we achieved EdU fluorescence labeling with the EdU Cell Proliferation Kit, and cell nucleuses were dyed with Hoechst solution (C1025, Beyotime) at room temperature in dark. The proliferating cells were stained green, whereas the nuclei of all cells were stained blue. The images of EdU-positive cells were captured at a magnification of 100× using an inverted fluorescence microscope (Olympus). Cell counting was conducted by ImageJ software. The percentage of EdU-positive cells were calculated by the following formula: EdU-positive rate = EdU-positive cell count/(EdU-positive cell count + EdU-negative cell count) × 100%.

### CCK8 cell proliferation assay

The AGS and MKN28 cells transfected with plasmids (2000–3000 cells/well) were cultivated in five 96-well plates with four replicate wells. Afterward, a CCK8 Cell Proliferation Kit (C0037, Beyotime) was utilized following the manufacturer’s suggestions. Thereafter, this study added CCK-8 solution to each well at 24/48/72/96 h, followed by 2-h incubation at 37°C. Then, each cell plate was assayed using a Varioskan LUX Multimode Microplate Reader (Thermo) at a wavelength of 450 nm. The experiment was performed independently in triplicate.

### Cell cycle and Cell apoptosis analysis

We adopted the Cell Cycle and Apoptosis Detection Kit (C1052, Beyotime) to measure cell cycle by flow cytometry (FCM). After staining using PI solution (500 μL) under 37°C for 30 min, we conducted FCM analysis on cells based on the FACSVerse flow cytometer (BD Biosciences). The percentages of cells within various phases of the cell cycle were calculated using FlowJo software (Treestar, Ashland, OR).

Apoptosis of AGS cells was measured through FCM using the Annexin V-APC/7‑AAD (Annexin V-Allophycocyanin/7-aminoactinomycin D (APC/7‑AAD)) Apoptosis kit (No.70-AP105-100, Multi Science, Hangzhou, China). Annexin V-FITC/PI (Annexin V-Fluorescein isothiocyanate/Propidium Iodide (FITC/PI)) Apoptosis Kit (No.70-AP101-100, Multi Science) was later utilized to stain MKN28 cells. In line with specific protocols, we incubated different cell lines using Annexin V‑APC, FITC, 7‑AAD, or PI in the dark centrifuge tubes for a 5-min period under ambient temperature. All procedures for FCM analysis were performed as described above.

### Transwell assay

The 24-well Transwell chambers (Corning, NY, USA) were adopted for performing Transwell invasion and migration experiments. The upper chamber was precoated with Matrigel (M8370, Solarbio) to assess the cell invasive ability. AGS and MKN28 cells were resuspended into a single-cell suspension in Ham’s F12 or RPMI-1640 medium containing 1% FBS, and then cells (3x10^4^ cells/well) were seeded into the upper chamber in triplicate. Subsequently, we added 100 ul Ham’s F12 or RPMI-1640 medium that contained 15% FBS (as the chemoattractant) in bottom chambers. After at least 24-h incubation, membrane fixation, and 1-h of 0.5% crystal violet-methanol staining were performed. Then, we eliminated those non-migratory cells within the top chambers gently using PBS and cotton swabs. Without Matrigel within the upper chamber, this work conducted Transwell assays for detecting migration of AGS and MKN28 cells. The subsequent steps were done as mentioned in the cell invasion assay section. We calculated cell numbers from four representative fields per membrane using light microscopy to determine the mean value in each field.

### Wound healing assay

The cell migration activity was performed in wound healing assay. Straight vertical and horizontal lines were scratched in the cell monolayers using a 200 μl pipette tip. After the initial scratch, the cells were allowed to migrate and images of the scratch width were taken at 0 h and 24 h, respectively. Then, the area occupied by migrating cells was measured by using ImageJ software. Cell mobility rate (%) = (original scratch area – 24 h scratch area)/original scratch area × 100. Each experiment consisted of three sets of replicates.

### Xenograft nude mouse models

Ten 4-week-old female SPF BALB/c nude mice were obtained at Nanjing Junke Biological Engineering Co. Ltd. (Nanjing, China). Each mouse was given subcutaneous injection of AGS cells stably transfected with shRNA-DDX56-LV (sh-DDX56-LV) and scramble-DDX56-LV (NC-DDX56-LV) via right flank. Under deep anesthesia, mice were euthanized through cervical dislocation after 29 days. We determined tumor volume below, tumor volume (mm^3^) = [length (mm) × width (mm) [2]] π/6. The use of animals was performed strictly according to the National Institutes of Health’s Guide for the Care and Use of Laboratory Animals [25].

### High-throughput sequencing technology

RNA-seq transcriptome high-throughput sequencing and subsequent bioinformatics analysis were performed by Sangon Biological Engineer Technology (Shanghai, China). In line with specific protocols, RNA‐seq library was established with the VAHTSTM mRNA-seq V2 Library Prep Kit for Illumina® (San Diego, CA, USA). Prior to high-throughput sequencing, libraries and their purity were validated on an Agilent Technologies 2100 Bioanalyzer (Santa Clara, CA, USA). We then conducted high-throughput sequencing with the Illumina HiSeq TM2500 Sequencing System. The sequencing output consisted of generating raw reads as the starting materials, so as to analyze the mRNA-seq data.

### Statistical analysis

Computation was conducted by SPSS22.0 (SPSS, Inc., Chicago, IL, USA) and GraphPad Prism software (GraphPad Prism Software, Inc., San Diego, CA). The ‘ggpubr’ package in R (https://cran.r-project.org/web/packages/ggpubr/index.html) was employed to perform statistical analyses and draw boxplots. The Encyclopedia of RNA Interactomes (ENCORI, https://starbase.sysu.edu.cn/index.php) [26] was utilized to predict the potential relationship of DDX56 expression with relevant cell cycle genes in STAD. GSEA (https://www.broadlnstitute.org/gsea/) was adopted to investigate the underlying mechanism of the expression of DDX56 in GC. We performed a student’s t-test for analyzing significant difference of 2 groups. Thereafter, we compared multiple groups by one-way or two-way ANOVA. Clinicopathological characteristics were detected through chi-square tests. P < 0.05 stood for statistical significance. All experiments described above were performed in triplicate.

## Results

The purpose of this study was to explore the potential role of DDX56 in gastric cancer. In vivo and in vitro experiments indicated that DDX56 was associated with GC cell proliferation, apoptosis, invasion and migration. The public databases and western blotting assay were chosen to verify the functional role of DDX56 and the expression changes of key signaling molecules.

### High expression of DDX56 in GC and its relation with poor prognosis

**Figure 1.**
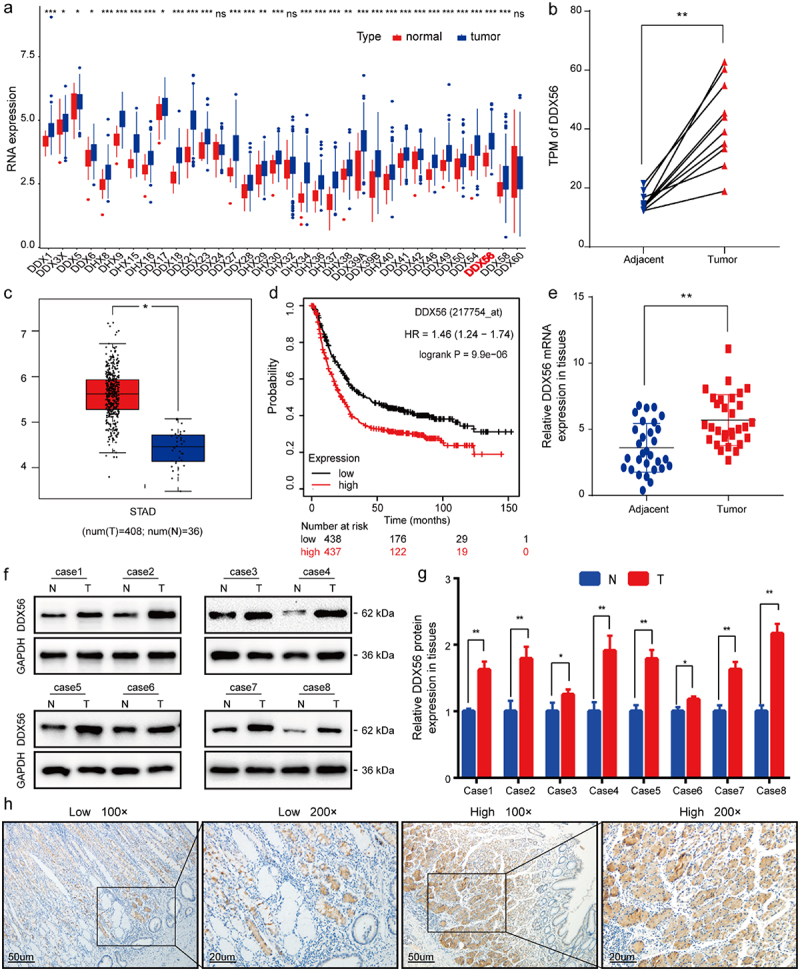
Identification and validation of a novel oncogenic DDX56 gene in human GC tissues. (a) The Box plot showing DDX family gene expression within GC tissues and normal gastric mucosa using ‘ggpubr’ package in R. (b) A total of 10 paired GC tissues were analyzed by high-throughput sequencing. RNA-seq data were reported as TPM to plot a dot line chart. (c) The Box plot presenting the DDX56 gene expression levels in GC and normal tissues from the TCGA-STAD project. (d) K-M curve showing OS according to different DDX56 expression levels using TCGA. (e) Relative DDX56 mRNA levels were compared between GC samples (n = 30) and normal gastric tissues (n = 30). (f, g) Relative expression levels of DDX56 protein in GC and adjacent normal tissues were normalized to GAPDH level. (h) IHC staining of DDX56 expression in human GC tissues and normal adjacent tissues. Asterisks stand for statistically significant differences: * P < 0.05, ** P < 0.01, *** P < 0.001, **** P < 0.0001. Abbreviations: TPM, Transcripts Per Million; STAD, stomach adenocarcinoma; N, normal; T, tumor.

According to our results, the DDX56 expression showed statistically significant difference in DDX family genes (Figure 1(a), p < 0.001). High-throughput sequencing (RNA-seq) results reflected that the TPM of DDX56 within GC samples increased compared with non-carcinoma samples (Figure 1(b), p < 0.01). The similar result was also observed in the TCGA-STAD cohort (Figure 1(c), p < 0.05). Furthermore, according to the Kaplan–Meier survival curve, GC cases with DDX56 up-regulation showed the worse outcome in comparison with those with DDX56 down-regulation (Figure 1(d), p < 0.0001). Subsequently, qRT-PCR and WB assays revealed that DDX56 expression increased within cancerous tissues compared with adjacent non-cancerous tissues in 30 pairs of GC patients (Figure 1(e-g), p < 0.05, P < 0.01). This study classified IHC staining sections as low or high group based on the score (Figure 1(h)). Clinical data and clinicopathological characteristics are detailed in Table 1. The relation of DDX56 expression with clinicopathological features was evaluated, revealing the relation of DDX56 with lymph node metastasis (P < 0.01). DDX56 protein levels were not significantly different in stage I–II compared with stage III–IV tumors according to IHC staining (Table 1).

**Table 1.** Correlation between immunohistochemistry staining intensity for DDX56 and clinicopathological features in 30 GC patients.

			DDX56 expression		
Characteristics		Case	Low	High	*p*-value
Age (years)					
<60		18	7	11	1.000
≥60		12	4	8	
Gender					
Male		19	7	12	1.000
Female		11	4	7	
Tumor size (cm)					
≥5		11	2	9	0.140
<5		19	9	10	
Tumor site					
Cardiac		3	1	2	1.000
Non-cardiac		27	10	17	
Differentiation					
PD		29	11	18	1.000
MD		1	0	1	
T grade					
T1 + T2		3	1	2	0.501
T3 + T4		27	10	17	
Lymph node metastasis					
Negative (N0)		8	6	2	0.028^#^
Positive (N1-N3)		22	5	17	
TNM stage					
I-II		7	5	2	0.068
III-IV		23	6	17	

PD, poorly differentiated; MD, moderately differentiated.

#: Significantly different at p < 0.05.

### Construction of DDX56 knockdown and overexpression GC cell models

**Figure 2.**
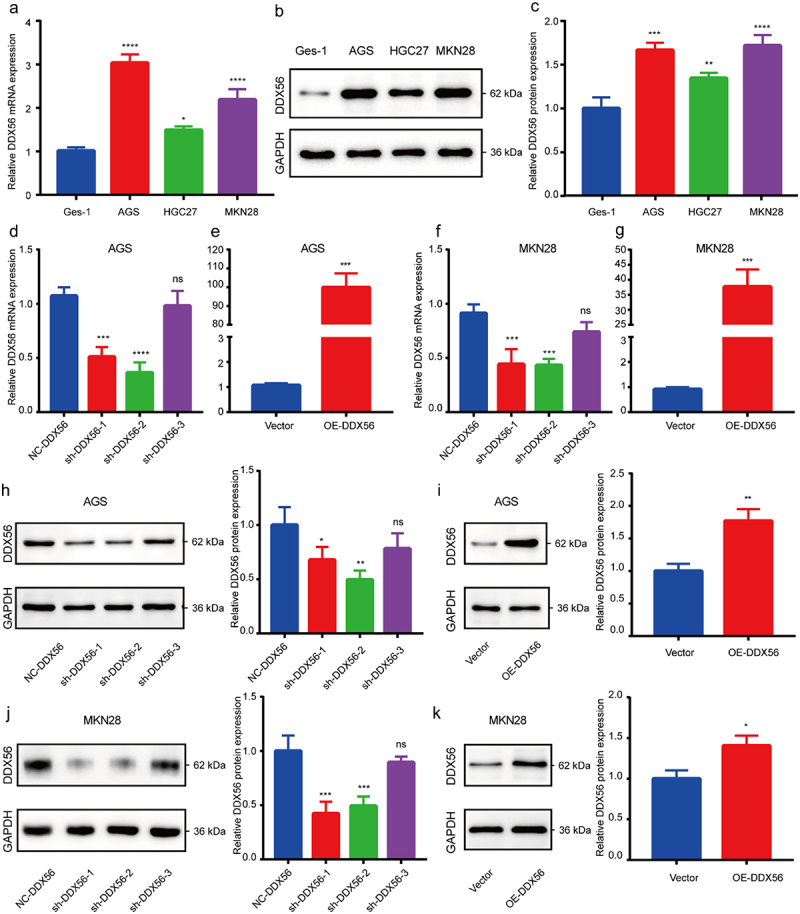
Construction of DDX56 knockdown and overexpression cell models. (a) qRT-PCR analysis on DDX56 levels within healthy gastric epithelium cells (Ges-1) and three GC cell lines (AGS, HGC27, MKN28). (b, c) WB assay on DDX56 expression within Ges-1, AGS, HGC27 and MKN28 cells. Densitometry showing the relative protein expression normalized to GAPDH. (d-g) Fold differences in mRNA expression of DDX56 were determined by qRT-PCR in AGS cells and MKN28 cells afte*r plasmid* transfection. (h-k) Relative DDX56 protein expression was detected by WB assays afte*r plasmid* transfection and quantitatively analyzed. Asterisks indicate statistical significance: * P < 0.05, ** P < 0.01, *** P < 0.001, **** P < 0.0001, NS, P > 0.05, not significant.

In AGS and MKN28 cells, DDX56 expression increased compared with that in HGC27 cells (Figure 2(a-c), p < 0.05, P < 0.01, P < 0.001, P < 0.0001). Therefore, we chose AGS and MKN28 cells in later analyses. The results of qRT-PCR and WB assays showed that the sh-DDX56-2 had a greater knockdown efficiency than other shRNAs (Figure 2(d-j), p < 0.05, P < 0.01, P < 0.001, P < 0.0001, NS not significant, P > 0.05). Similarly, we conducted qRT-PCR and Cell monolayers assays to verify the successful construction of DDX56 overexpression (Figure 2(e-k), p < 0.05, P < 0.01, P < 0.001).

### Knockdown of DDX56 inhibits cell proliferation and arrests in the G1/S phase

**Figure 3.**
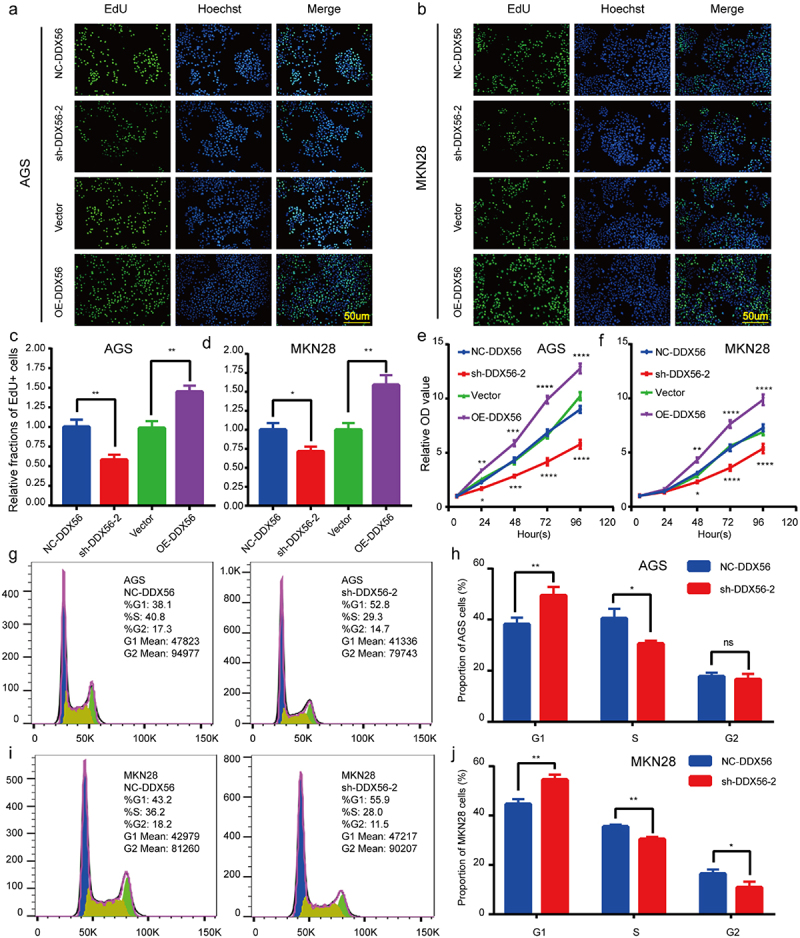
DDX56 is associated with GC cell proliferation. (a, c) EdU assays and quantitative results for AGS cells after transfection with plasmid. (b, d) EdU assays and quantitative measurements for MKN28 cells after transfection with plasmid. (e) Proliferation of plasmid-transfected AGS cells was measured by CCK8 assay. (f) Cell viability of plasmid-transfected MKN28 cells was determined using CCK8 assay. (g-j) Representative images and quantitative flow cytometry results for cell cycle arrest in AGS and MKN28 cells transfected with NC-DDX56 plasmid and sh-DDX56-2 plasmid. Abbreviations: CCK-8, Cell Counting Kit-8. Asterisks indicate statistical significance: * P < 0.05, ** P < 0.01, *** P < 0.001, **** P < 0.0001, NS, P > 0.05, not significant.

Based on results of EdU assay and CCK8 assay, the cell proliferation of sh-DDX56-2 group was inhibited compared with that of NC-DDX56 group. By contrast, cell proliferation was obviously promoted in cells of OE-DDX56 group relative to those of Vector group (Figure 3(a-f), p < 0.05). To better examine DDX56s impact on the proliferation of AGS and MKN28 cells, we conducted FCM to detect cell cycle. As a result, G1 phase cell proportion increased when DDX56 was silenced, whereas the proportion of S phase cells decreased (Figure 3(g-j), p < 0.05). Besides, the proportion of G2 phase AGS cells harboring the alteration exhibited no statistically significant difference (Figure 3(j)).

### DDX56 stimulates migration and invasion of GC cells

**Figure 4.**
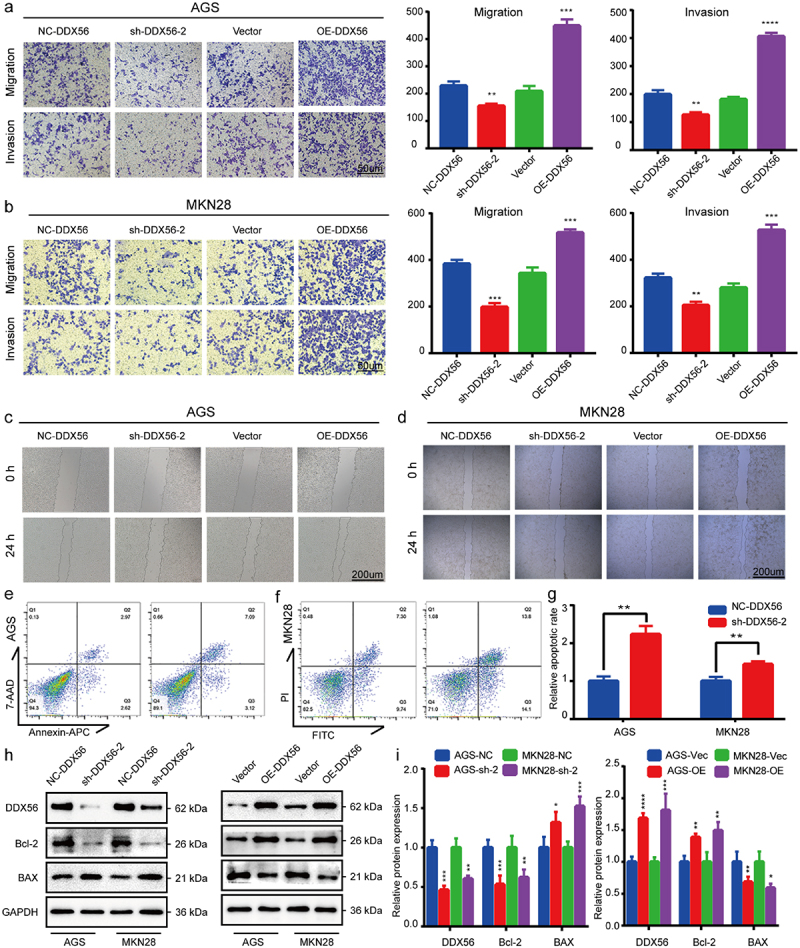
DDX56 is associated with cell apoptosis, invasion and migration. (a) Representative photographs of Transwell assays and quantitative analysis in AGS cells transfected with plasmids. (b) Representative images and quantitative analysis of Transwell assays in MKN28 cells after transfection with plasmid. (c, d) AGS and MKN28 cell migration rates after transfection with plasmids for 0 h and 24 h assessed by wound healing assay. (e) Apoptosis of AGS cells transfected with NC-DDX56 plasmid and sh-DDX56-2 plasmid revealed by FCM analysis. (f) FCM was conducted to show the apoptosis of plasmid-transfected MKN28 cells. (g) Bar plot showing the relative fraction of apoptosis cells. (h) The respective ratios of DDX56, Bcl-2 and BAX protein levels to GAPDH as an internal control. (i) The bar graphs representing relative protein expression levels in AGS and MKN28 cells. Asterisks indicate statistical significance: * P < 0.05, ** P < 0.01, *** P < 0.001, **** P < 0.0001, NS, P > 0.05, not significant.

Results of Transwell assay demonstrated that DDX56 knockdown inhibited AGS and MKN28 cell migration and invasion, while DDX56 overexpression had opposite effects (Figure 4(a-b), p < 0.01). This study conducted scratch assay to better determine the action of DDX56 in cell migration ability. As a result, DDX56 knockdown led to reduced cell migration of sh-DDX56-2 groups. At the same time, scratch width narrowed significantly in OE-DDX56 groups (Figure 4(c-d), p < 0.05) (The quantitative results of wound healing assay are shown in the supplementary document).

### Knockdown of DDX56 promotes cell apoptosis

The FCM apoptosis assay indicated that DDX56 knockdown increased the number of apoptosis cells compared with NC-DDX56 groups (Figure 4(e-g), p < 0.01). Accordingly, results of WB assay verified this observation in protein levels. Knockdown of DDX56-induced cell apoptosis by down-regulating the expression of Bcl-2 protein and up-regulating that of BAX protein (Figure 4(h-i), p < 0.05). Overexpression of DDX56 increased Bcl-2 protein expression but reduced BAX expression (Figure 4(h-i), p < 0.05).

### Pathway enrichment

**Figure 5.**
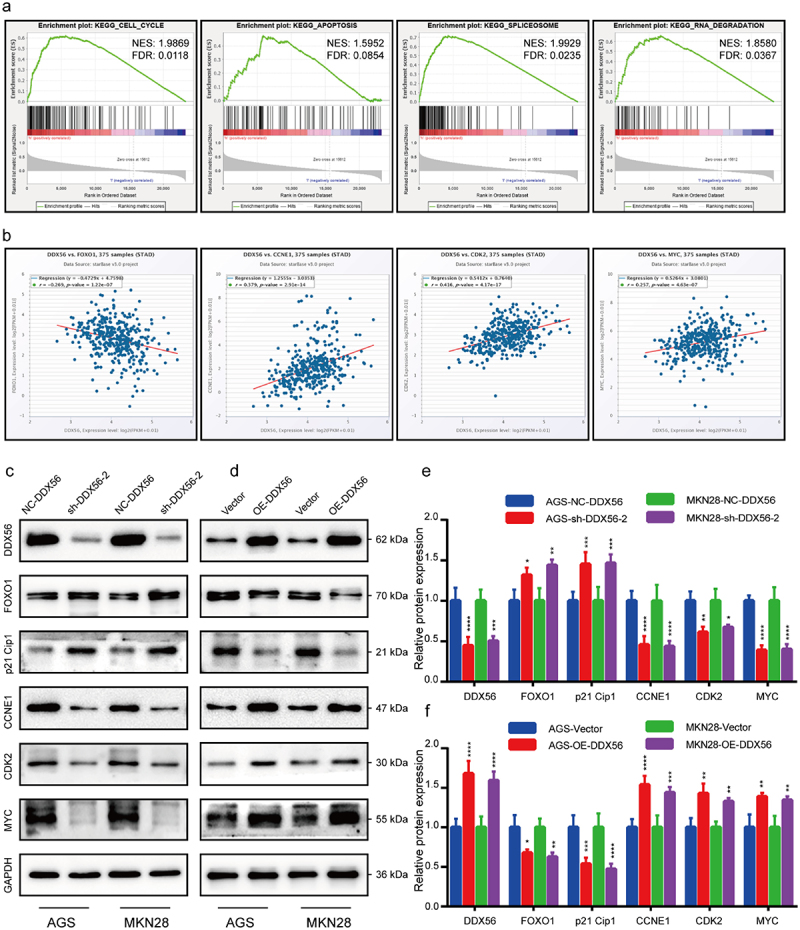
GSEA enrichment analysis and ENCORI reveal that cell proliferation is closely related to DDX56. (a) GSEA demonstrated that the genes involved in cell cycle, apoptosis, spliceosome and RNA degradation pathways were more significantly correlated with DDX56. (b) The results of correlation analysis of DDX56 with FOXO1, CCNE1, CDK2 and MYC were predicted by the ENCORI database. (c, d) The protein expression levels of DDX56, FOXO1, p21 Cip1, CCNE1, CDK2, MYC and GAPDH in both AGS and MKN28 cells were determined by WB assay. (e, f) Bar graphs indicating the relative protein levels (GAPDH as reference) in WB analysis. Asterisks stand for statistically significant differences: * P < 0.05, ** P < 0.01, *** P < 0.001, **** P < 0.0001.

According to GESA, DDX56 up-regulation showed strong correlation with genes related to the cell cycle, apoptosis, spliceosome, and RNA degradation signaling pathways (Figure 5(a), |NES| > 1, P < 0.05, FDR q-val < 0.25). Thereafter, ENCORI predicted the mRNA interaction analysis on the above pathways for DDX56. As a result, FOXO1 was negatively correlated with DDX56, but positively related to CCNE1 (cyclin E1), CDK2, and MYC (c-Myc) (Figure 5(b), p < 0.0001). As revealed by WB assay, the expression of CCNE1, CDK2, and MYC proteins decreased, while that of FOXO1 and p21 Cip1 proteins increased by DDX56 knockdown (Figure 5(c, e), p < 0.05). In DDX56 overexpression cells, the expression of CCNE1, CDK2, and MYC proteins increased, while that of FOXO1 and p21 Cip1 proteins decreased (Figure 5(d-f), p < 0.05).

### DDX56 modulates tumor growth in vivo

**Figure 6.**
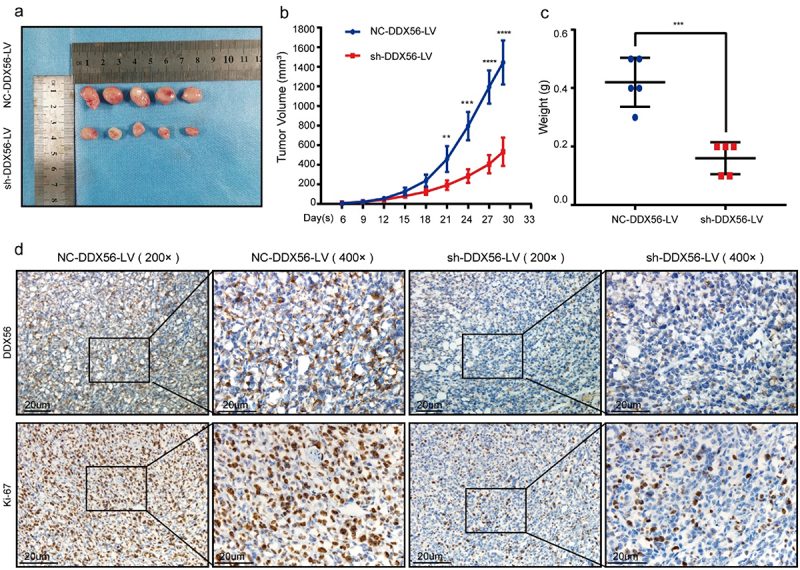
The effects of DDX56 modulation on the growth of xenografts in nude mice. (a) Typical photographs showing xenograft-bearing BALB/c nude mice (n =  5 per group). (b) Growth curves for the xenografts within nude mice. (c) Tumor weights of subcutaneous xenografts model in BALB/c nude mice were measured. (d) Expression of DDX56 protein and Ki-67 protein in tumor tissues of tumor-bearing nude mice (IHC staining, 200x and 400x).

Knockdown of DDX56 significantly inhibited the subcutaneous tumors of AGS cells, which was consistent with the results in vitro. Besides, the results of subcutaneous xenograft tumors indicated that the tumor volume and mean weigh of NC-DDX56-LV group were significantly larger than those of sh-DDX56-LV group (Figure 6(a-c), p < 0.01). IHC experiments verified that the DDX56 protein and Ki-67 protein expression in nude mice of sh-DDX56-LV group was lower than that of NC-DDX56-LV group (Figure 6(d)).

## Discussion

As the hallmarks of cancer, evasion of growth suppressors and persistence of proliferation signal transduction remain the two parts of diverse cancers [27]. Increasing evidence has demonstrated that the DDX family regulates cell proliferation, which in turn affects tumor progression. DDX46 silencing arrested cell cycle at G1 phase in esophageal squamous cell carcinoma (ESCC) cell lines [28]. Moreover, the knockdown of DDX3 [29] and DHX33 [30,31] inhibited cell cycle progression by blocking the entry into S phase of cancer cells. However, some studies indicate that DDX5, DDX21, and DDX3X exert tumor suppressor function in different cancers, such as hepatocellular carcinoma (HCC) [32], breast cancer (BC) [33] and colorectal cancer (CRC) [34]. In this study, we first identified the differential DDX56 mRNA expression between GC and matched non-carcinoma tissue samples by high-throughput sequencing. The boxplot showed that DDX56 showed significant difference in DDX family genes in STAD datasets. Subsequently, we investigated 30 GC and paired normal samples. As a result, DDX56 levels increased within GC samples than paired non-carcinoma samples via qRT-PCR and WB assays. Images of IHC sections revealed that DDX56 protein was mainly expressed in the lamina propria of the stomach mucosa. The clinicopathological data indicated that the high levels of DDX56 were related to lymph node metastasis. Similarly, the results of wound healing and Transwell assays suggested that silencing DDX56 impaired GC cell invasion and migration. Consistently, Kaplan–Meier survival curve demonstrated that the high DDX56 expression was correlated with the poor prognosis of patients with GC in the current study. These results arouse our great interest.

DDX56 is located on chromosome 7 and regulates diverse RNA metabolic processes, including transcription, translation, ribosome assembly, ribosome maturation, pre-mRNA splicing, and mRNA degradation [7,35]. According to results of EdU and CCK8 assays, knockdown of DDX56 suppressed GC cell proliferation, while overexpression of DDX56 promoted proliferation. Furthermore, the suppression of tumor growth in DDX56 knockdown cells was confirmed in nude mice. Several studies have pointed out that DDX3 promotes G1/S transition by promoting the translation of cyclin E1 and up-regulating the expression of CDK2 [29,36]. DDX41 can bind to the 30 UTR of p21 Cip1 mRNA to inhibit its translation under basal and stress conditions [37]. DDX56 shares common structures with the DDX family members, and thus it may also regulate the process of cell cycle [6]. The results of GSEA pathway enrichment analysis indicated that DDX56 was strongly correlated with the Apoptosis and Cell cycle pathways. To better understand DDX56’s effect on the cell cycle, FCM was conducted. As a result, down-regulating DDX56 level enhanced G1-phase cell percentage, but reduced S-phase cell percentage. Similarly, WB results revealed that down-regulating the protein level of DDX56 increased the expression of p21 Cip1 protein, but impaired that of cyclin E1, CDK2, and c-Myc. C-Myc can be accumulated within GC, CRC, BC, and lung cancer (LC), and it increases the expression of numerous pro-proliferation genes, like transcriptional cell apoptosis [38]. In LC, pathway enrichment analysis indicated that c-Myc signaling is positively associated with DDX11, DDX55, and DDX56 [39]. The above results suggest that DDX56 may subsequently affect cell proliferation by regulating the cell cycle G1/S checkpoint. It has been extensively accepted that FOXO1 is one of the critical molecules regulating G1/S phase transition in tumor cells [40,41]. FOXO1’s anti-cancer role is associated with the regulation of critical genes related to cell cycle arrest and cell apoptosis such as p21 Cip1 [16]. In this study, based on WB assay, down-regulating DDX56 protein expression increased FOXO1 protein expression in AGS and MKN28 cells. By contrast, DDX56 overexpression suppressed FOXO1 expression. These results suggested that DDX56 knockdown arrest cell cycle at G1-S phase through increasing FOXO1 expression. Furthermore, we found that DDX56 down-regulation led to elevated expression of FOXO1 and BAX proteins, but down-regulated expression of Bcl-2 protein in AGS and MKN28 cells according to WB assay. Meanwhile, using FCM cell apoptosis assay, percentages of early apoptotic combined with late apoptotic cells increased by DDX56 knockdown. Consistently, an analysis based on fluorescence-activated cell sorting (FACS) indicates that, the apoptosis rates of human colon cancer cells are higher in the presence of FOXO1 than in FOXO1 knockdown cells [18]. Additionally, Guan *et al*. discovered that a high dose of PPIs induced nuclear translocation of FOXO1, thus mediating apoptosis of GC [23].

However, some shortcomings should be noted in this study. The sample size of the patient models was relatively small in a single center. Although DDX56 is enriched primarily in the nucleolus, it disperses throughout the cytoplasm upon treatment with cytotoxic agents [42]. This phenotype predicts that DDX56 may have different functions in different cellular organelles. Future studies are needed to elucidate DDX562019;s distribution in GC cells.

## Conclusion

In conclusion, this study provides comprehensive evidence for DDX56 as a pro-cancer agent and a biomarker for gastric cancer prognosis. DDX56 overexpression was detected within GC cells and tissues. Gain- and loss-of-function studies suggested that DDX56 promotes gastric cancer progression by regulating cell proliferation, apoptosis, invasion and migration functions. Moreover, the results of the study further elucidate the carcinogenic effects of the DDX56 via the FOXO1/p21 Cip1/c-Myc signaling pathway. Therefore, DDX56-FOXO1 signaling pathway may be considered to be the possible anti-GC therapeutic target.

## Supplementary Material

Supplemental Material

## References

[1] Thrift AP, El-Serag HB. Burden of gastric cancer. Clin Gastroenterol Hepatol. 2020;18(3):534–542. 31362118 10.1016/j.cgh.2019.07.045 PMC8859863

[2] Arnold M, Abnet CC, Neale RE, et al. Global burden of 5 major types of gastrointestinal cancer gastroenterology. Gastroenterology. 2020;159(1):335–349 e15. 32247694 10.1053/j.gastro.2020.02.068 PMC8630546

[3] Nie Y, Wu K, Yu J, et al. A global burden of gastric cancer: the major impact of China. Expert Rev Gastroenterol Hepatol. 2017;11(7):651–661. 28351219 10.1080/17474124.2017.1312342

[4] Seeneevassen L, Bessede E, Megraud F, et al. Gastric cancer: advances in carcinogenesis research and new therapeutic strategies. Int J Mol Sci. 2021;22(7):3418. 33810350 10.3390/ijms22073418 PMC8037554

[5] Sergeeva O, Zatsepin T. RNA helicases as shadow modulators of cell cycle progression. Int J Mol Sci. 2021;22(6):2984. 33804185 10.3390/ijms22062984 PMC8001981

[6] Linder P, Jankowsky E. From unwinding to clamping - the DEAD box RNA helicase family. Nat Rev Mol Cell Biol. 2011;12(8):505–516. 21779027 10.1038/nrm3154

[7] Zhang L, Li X. DEAD-box RNA helicases in cell cycle control and clinical therapy cells. Cells. 2021;10(6):1540. 34207140 10.3390/cells10061540 PMC8234093

[8] Du C, Li DQ, Li N, et al. DDX5 promotes gastric cancer cell proliferation in vitro and in vivo through mTOR signaling pathway Sci Rep. Scientific Reports. 2017;7:42876. 28216662 10.1038/srep42876 PMC5316961

[9] Zhou J, Yong WP, Yap CS, et al. An integrative approach identified genes associated with drug response in gastric cancer. Carcinogenesis. 2015;36(4):441–451. 25742747 10.1093/carcin/bgv014

[10] Zhang T, Ma Z, Liu L, et al. DDX39 promotes hepatocellular carcinoma growth and metastasis through activating Wnt/beta-catenin pathway cell death dis. Cell Death & Disease. 2018;9(6):675. 29867138 10.1038/s41419-018-0591-0 PMC5986742

[11] Wang Q, Ye Y, Lin R, et al. Analysis of the expression, function, prognosis and co-expression genes of DDX20 in gastric cancer comput struct. Biotechnol J. 2020;18:2453–2462. 10.1016/j.csbj.2020.09.002 PMC7509587 33005307

[12] Huang Y, Wang C, Li K, et al. Death-associated protein kinase 1 suppresses hepatocellular carcinoma cell migration and invasion by upregulation of DEAD-box helicase 20 Cancer Sci. Cancer Science. 2020;111(8):2803–2813. 32449268 10.1111/cas.14499 PMC7419049

[13] Kouyama Y, Masuda T, Fujii A, et al. Oncogenic splicing abnormalities induced by DEAD-Box Helicase 56 amplification in colorectal cancer cancer sci. Cancer Science. 2019;110(10):3132–3144. 31390121 10.1111/cas.14163 PMC6778637

[14] Zhu C, Zhang X, Kourkoumelis N, et al. Integrated analysis of DEAD-box helicase 56: a potential oncogene in osteosarcoma front bioeng biotechnol. Frontiers in Bioengineering and Biotechnology. 2020;8:588. 32671031 10.3389/fbioe.2020.00588 PMC7332757

[15] Wu Q, Luo X, Terp MG, et al. DDX56 modulates post-transcriptional Wnt signaling through miRNAs and is associated with early recurrence in squamous cell lung carcinoma mol cancer. Molecular Cancer. 2021;20(1):108. 34446021 10.1186/s12943-021-01403-w PMC8393456

[16] Laissue P. The forkhead-box family of transcription factors: key molecular players in colorectal cancer pathogenesis. Mol Cancer 2019;18(1):5. 30621735 10.1186/s12943-019-0938-x PMC6325735

[17] Peng S, Li W, Hou N, et al. A Review of FoxO1-regulated metabolic diseases and related drug discoveries. Cells.2020;9(1):184. 10.3390/cells9010184 PMC7016779 31936903

[18] Chae YC, Kim JY, Park JW, et al. FOXO1 degradation via G9a-mediated methylation promotes cell proliferation in colon cancer Nucleic Acids Res. 2019;47(4):1692–1705. 30535125 10.1093/nar/gky1230 PMC6393239

[19] Xing YQ, Li A, Yang Y, et al. The regulation of FOXO1 and its role in disease progression. Life Sci. 2018;193:124–131. 29158051 10.1016/j.lfs.2017.11.030

[20] Pan S, Deng Y, Fu J, et al. Decreased expression of ARHGAP15 promotes the development of colorectal cancer through PTEN/AKT/FOXO1 axis cell death dis. Cell Death & Disease. 2018;9(6):673. 29867200 10.1038/s41419-018-0707-6 PMC5986807

[21] Hume S, Dianov GL, Ramadan K. A unified model for the G1/S cell cycle transition nucleic acids res. Nucleic Acids Research. 2020;48(22):12483–12501. 33166394 10.1093/nar/gkaa1002 PMC7736809

[22] Wang H, Shi H. Megestrol acetate drives endometrial carcinoma cell senescence via interacting with progesterone receptor B/FOXO1 axis. Exp Biol Med (Maywood). 2021;246(21):2307–2316. 34233525 10.1177/15353702211026566 PMC8581832

[23] Guan XW, Zhao F, Wang JY, et al. Tumor microenvironment interruption: a novel anti-cancer mechanism of Proton-pump inhibitor in gastric cancer by suppressing the release of microRNA-carrying exosomes. Am J Cancer Res. 2017;7(9):1913–1925. 28979813 PMC5622225

[24] Zhao Z, Li C, Xi H, et al. Curcumin induces apoptosis in pancreatic cancer cells through the induction of forkhead box O1 and inhibition of the PI3K/Akt pathway mol med rep. Molecular Medicine Reports. 2015;12(4):5415–5422. 26166196 10.3892/mmr.2015.4060

[25] Guide for the Care and Use of Laboratory Animals.pdf 2011.

[26] Li JH, Liu S, Zhou H, et al. starBase v2.0: decoding miRNA-ceRNA, miRNA-ncRNA and protein-RNA interaction networks from large-scale CLIP-Seq data Nucleic Acids Res. Nucleic Acids Research. 2014;42(Database issue):D92–7. 24297251 10.1093/nar/gkt1248 PMC3964941

[27] Hanahan D, Weinberg RA. Hallmarks of cancer: the next generation. Cell. 2011;144(5):646–674. 21376230 10.1016/j.cell.2011.02.013

[28] Chen L, Xu M, Zhong W, et al. Knockdown of DDX46 suppresses the proliferation and invasion of gastric cancer through inactivating Akt/GSK-3beta/beta-catenin pathway Exp Cell Res. Experimental Cell Research. 2021;399(1):112448. 33347858 10.1016/j.yexcr.2020.112448

[29] Cannizzaro E, Bannister AJ, Han N, et al. DDX3X RNA helicase affects breast cancer cell cycle progression by regulating expression of KLF4 FEBS Lett. FEBS Letters. 2018;592(13):2308–2322. 29782654 10.1002/1873-3468.13106 PMC6100109

[30] Wang H, Yu J, Wang X, et al. The RNA helicase DHX33 is required for cancer cell proliferation in human glioblastoma and confers resistance to PI3K/mTOR inhibition Cell Signal. Cellular Signalling. 2019;54:170–178. 30552990 10.1016/j.cellsig.2018.12.005

[31] Wang J, Feng W, Yuan Z, et al. DHX33 Interacts with AP-2beta to regulate Bcl-2 gene expression and promote cancer cell survival. Mol Cell Biol. 2019;39(17):e00017–19. 31182639 10.1128/MCB.00017-19 PMC6692123

[32] Liu X, Meng L, Li X, et al. Regulation of FN1 degradation by the p62/SQSTM1-dependent autophagy-lysosome pathway in HNSCC. Int J Oral Sci. 2020;12(1):34. 33318468 10.1038/s41368-020-00101-5 PMC7736930

[33] Zhang H, Zhang Y, Chen C, et al. A double-negative feedback loop between DEAD-box protein DDX21 and Snail regulates epithelial-mesenchymal transition and metastasis in breast cancer Cancer Lett. Cancer Letters. 2018;437:67–78. 30165191 10.1016/j.canlet.2018.08.021

[34] Lin TC. DDX3X multifunctionally modulates tumor progression and serves as a prognostic indicator to predict cancer outcomes. Int J Mol Sci. 2019;21(1):281. 10.3390/ijms21010281 PMC6982152 31906196

[35] Wang J, Liu J, Ye M, et al. Ddx56 maintains proliferation of mouse embryonic stem cells via ribosome assembly and interaction with the Oct4/Sox2 complex. Stem Cell Res Ther. 2020;11(1):314. 32703285 10.1186/s13287-020-01800-w PMC7376950

[36] Wu DW, Liu WS, Wang J, et al. Reduced p21(WAF1/CIP1) via alteration of p53-DDX3 pathway is associated with poor relapse-free survival in early-stage human papillomavirus-associated lung cancer. Clin Cancer Res. 2011;17(7):1895–1905. 21325288 10.1158/1078-0432.CCR-10-2316

[37] Peters D, Radine C, Reese A, et al. The DEAD-box RNA helicase DDX41 is a novel repressor of p21(WAF1/CIP1) mRNA translation. J Biol Chem. 2017;292(20):8331–8341. 28348086 10.1074/jbc.M116.772327 PMC5437239

[38] Sarosiek KA, Fraser C, Muthalagu N, et al. Developmental regulation of mitochondrial apoptosis by c-Myc governs age- and tissue-specific sensitivity to cancer therapeutics. Cancer Cell. 2017;31(1):142–156. 28017613 10.1016/j.ccell.2016.11.011 PMC5363285

[39] Cui Y, Hunt A, Li Z, et al. Lead DEAD/H box helicase biomarkers with the therapeutic potential identified by integrated bioinformatic approaches in lung cancer Comput Struct. Biotechnol J. 2021;19:261–278. 10.1016/j.csbj.2020.12.007 PMC7779375 33425256

[40] Yang S, Pang L, Dai W, et al. Role of forkhead box O proteins in hepatocellular carcinoma biology and progression (Review. Front Oncol. 2021;11:667730. 34123834 10.3389/fonc.2021.667730 PMC8190381

[41] Jie M, Wu Y, Gao M, et al. CircMRPS35 suppresses gastric cancer progression via recruiting KAT7 to govern histone modification mol cancer. Molecular Cancer. 2020;19(1):56. 32164722 10.1186/s12943-020-01160-2 PMC7066857

[42] Pryszlak M, Wiggans M, Chen X, et al. The DEAD-box helicase DDX56 is a conserved stemness regulator in normal and cancer stem cells Cell Rep. Cell Reports. 2021;34(13):108903. 33789112 10.1016/j.celrep.2021.108903

