# Supplementary material for: DEAD-box helicase 56 functions as an oncogene promote cell proliferation and invasion in gastric cancer via the FOXO1/p21 Cip1/c-Myc signaling pathway
Source: Bioengineered. 2022 Jun 19;13(5):13970–85. doi: 10.1080/21655979.2022.2084235 (PMC9275944; doi:10.1080/21655979.2022.2084235)
Supplement: Supplemental Material [file MKN28 Wound healing Quantitative Histogram.pdf › Supplementary data for reviewer 1/MKN28 Wound healing Quantitative Histogram.pdf]

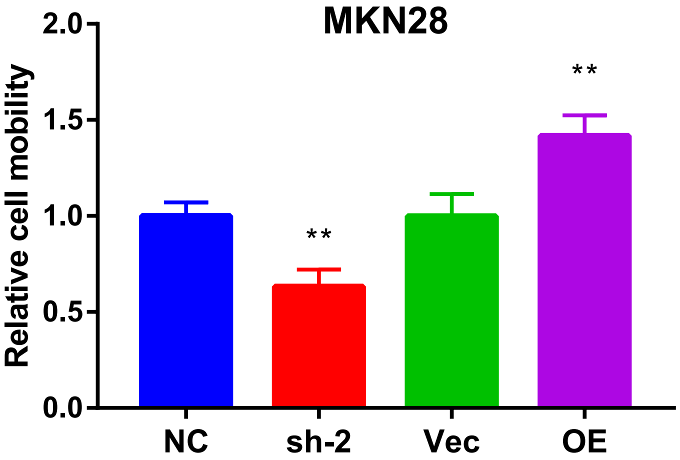
MKN28
Relative cell mobility
2.0
1.5
1.0
0.5
0.0
**
**
NC
sh-2
Vec
OE
